# 2D MHD Mixed Convection in a Zigzag Trapezoidal Thermal Energy Storage System Using NEPCM

**DOI:** 10.3390/nano12193270

**Published:** 2022-09-20

**Authors:** Aissa Abderrahmane, Obai Younis, Mohammad Al-Khaleel, Houssem Laidoudi, Nevzat Akkurt, Kamel Guedri, Riadh Marzouki

**Affiliations:** 1 Laboratoire de Physique Quantique de la Matière et Modélisation Mathématique (LPQ3M), University of Mascara, Mascara 29000, Algeria; a.aissa@univ-mascara.dz; 2 Department of Mechanical Engineering, College of Engineering in Wadi Addwasir, Prince Sattam Bin Abdulaziz University, Al-Kharj 11942, Saudi Arabia; oubeytaha@hotmail.com; 3 Department of Mathematics, Khalifa University, Abu Dhabi 127788, United Arab Emirates; 4 Department of Mathematics, Yarmouk University, Irbid 21163, Jordan; 5 Laboratory of Sciences and Marine Engineering (LSIM), Oran 31000, Algeria; houssem.laidoudi@univ-usto.dz; 6 Department of Mechanical Engineering, Munzur University, 62000 Tunceli, Turkey; nakkurt@munzur.edu.tr; 7 Mechanical Engineering Department, College of Engineering and Islamic Architecture, Umm Al-Qura University, P.O. Box 5555, Makkah 21955, Saudi Arabia; kmguedri@uqu.edu.sa; 8 Chemistry Department, College of Science, King Khalid University, Abha 61413, Saudi Arabia; rmarzouki@kku.edu.sa; 9 Chemistry Department, Faculty of Sciences of Sfax, University of Sfax, Sfax 3038, Tunisia

**Keywords:** magneto-hydrodynamics, NEPCM, 2D flow, melting process, GFEM, zigzag trapezoidal vessels

## Abstract

In a magnetic field, two-dimensional (2D) mixed convection is investigated within a zigzagged trapezoidal chamber. The lower side of the trapezoidal chamber is irregular, in particular, a zigzagged wall with different zigzag numbers N. The fluid particles move in the room due to the motion of the upper wall, while the porosity-enthalpy approach represents the melting process. The thermal parameters of the fluid are enhanced by what is called a nano-encapsulated phase change material (NEPCM) consisting of polyurethane as the shell and a nonadecane as the core, while water is used as the base fluid. In order to treat the governing equations, the well-known Galerkin finite element method (GFEM) is applied. In addition, the heat transfer (HT) and the dynamic behavior of flow are treated in terms of average Nusselt number and contours of streamlines. The main results show that the melt band curve of heat capacity behaves almost parabolically at smaller values of Reynolds number (Re) and larger values of Hartmann number (Ha). Moreover, minimizing the zigzag number is better in order to obtain a higher heat transfer rate.

## 1. Introduction

Phase-change materials (PCM) in thermal systems are gaining tremendous attraction for energy storage systems, heat exchangers, air-conditioning systems, green buildings, etc. The advantage of the PCM is that the energy is saved in the materials as latent energy during the phase transition process, resulting in the temperature being almost constant in the system. This process is also referred to as latent heat storage material, which is carried out in evaporators in cooling systems [1]. From the literature, it has been found that the PC properties of the different materials vary due to temperature variation. On behalf of the construction of the multiscale structure, the PCMs are categorized as organic, inorganic, and eutectics [2]. Cui et al. [3] reviewed the PCM to understand its application in building better. They found the best phase change material applications when the room air temperature was as low as 4.2 °C. Aftab et al. [4] used PCM-integrated latent heat storage systems for sustainable energy advancements. Choudhari et al. [5] employed a fin structure to develop a PCM battery thermal management system. Numerous pieces of research have been communicated using the PCM to upgrade the battery thermal management systems [6,7,8,9,10].

It is challenging to implement PCM in a two-phase state, in particular, force convection heat transfer in the solid-liquid phase. Miansari et al. [11] addressed the fluid motion in the melting process and illustrated that the fluid motion is high in the thicker tank, while motion became slower in the thinner tank. Hosseinizadeh et al. [12] encapsulated nano-enhanced phase change materials (NEPCMs) inside the core and shell of paraffin. They found that the NEPCM has a higher impact when used in the energy storage system compared to PCM. Alehosseini et al. [13] illustrated that NEPCMs have thermal and mechanical stability, which make better phase-change materials. Some researchers have seen the thermal properties of the fluid while using NEPCM and found that the heat transfer rate was enhanced by 23.6% [14]. Ghalambaz et al. [15,16] simulated the mixed and natural convection heat transfer through an inclined porous cavity in the presence of NEPCM. Nadezhda et al. [17] investigated the entropy generation of the partially triangular open cavity in the presence of natural convection of a nanofluid. Seyf et al. [18] discussed the heat transfer in the microtube by using the phase-change heat transfer of NEPCMs. For the phase changer, many studies have extensively targeted heat transfer in tubes, micro conduits, and ducts. Varol [19] and Alshuraiaan [20] investigated the free convective heat transfer in a trapezoidal cavity without considering the NEPCM. Hossain et al. [21] investigated the magnetohydrodynamic (MHD) natural convective flow in a trapezoidal cavity filled by porous medium using the Galerkin weighted residual numerical approach (GWRNA). They obtained strong convection flow for high Ra (Rayleigh number) and Da (Darcy number) at Ha = 50 (Hartmann number). In the present model, we have considered the MHD mixed convection in the trapezoidal cavity for the dispersion of chemical contaminants through the fluids flow. Typically, cavities are carried out in industries such as triangular, square, trapezoidal, etc.

Khalil et al. [22] optimized the MHD free convective in the porous trapezoidal cavity using a multi-objective optimum technique. They found that heat transfer and energy enhancements (HTE and EE) are increased as the number of bottom wall waves increases. Hussain et al. [23] studied the entropy generation of natural convective flow inside the sinusoidal corrugated in the presence of the magnetic field. Extensive research and experimental works have been reported on both mixed and natural convective heat transfer in different cavities under different physical effects for different applications [24,25,26,27,28,29,30]. Few stabilization studies have been introduced for the heat transfer process in the cavities models. Galerkin Finite Element (GFE) is one of the most useful methods evolved from the FEM. For illustration, Chen et al. [31] used a class of the Galerkin method in order to analyze the two-dimensional natural convective porous medium flow. Al-Kouz et al. [32] studied entropy optimization of MHD convection in a cavity of odd shape using the GFE method. Generally, the usage of NEPCM in heat transfer applications results in enhancing heart transfer rates; however, special attention must be paid regarding nanoparticle accumulation as it would result in negative impacts, as reported by Qiu et al. [33] and Feng et al. [34].

According to the above literature survey, no literature has been reported regarding the MHD mixed convective flow within a trapezoidal cavity using the GFE method. In the present article, NEPCM is considered in the zigzag trapezoidal thermal storage system in order to enhance heat transfer and energy storage. The Galerkin Finite Element (GFEM) is applied to simulate the model’s numerical solutions. In addition, a comparative study of the heat transfer and the dynamic behavior of flow in terms of average Nusselt number and contours of streamlines has been presented. The outcomes of the model will be applicable in various energy systems, particularly for the storage of energy using the NEPCMs.

The current numerical work aims to respond to the following queries in the problem:

1. Does the velocity of the flow increase with Re along with the temperature distribution?
2. What is the role of the zigzag number on the flow velocity and the heat capacity?
3. Does increasing the value of Ha affect the heat transfer inside the cavity?

## 2. Formulation, Properties, and Problem Settings

**Figure 1:**
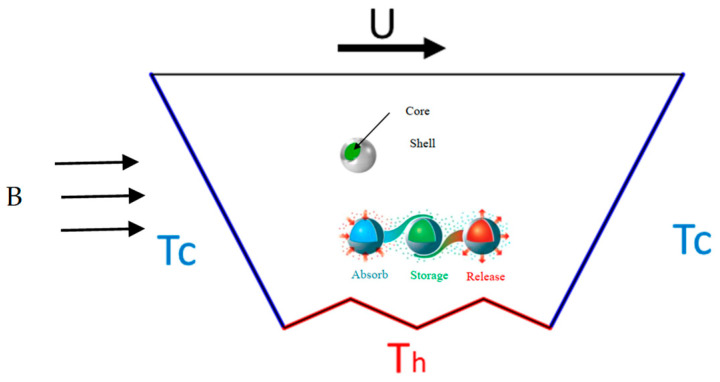
Physical model geometry.

The geometry consists of a trapezoidal prism cavity (see Figure 1). The upper cavity wall is moving at a constant velocity of U, while the rest of the walls are stationary. Mixed convection of liquid-NEPCM takes place in the cavity due to movement of the upper wall (forced convection) and the temperature difference between side walls and the lower zigzag wall (natural convection). The side walls of the chamber have a constant temperature of T_c_, whereas the temperature of the bottom zigzag wall is given by a constant temperature of T_h_. The PCM serves as the core of the nano-encapsulated PCM, while the encapsulating material serves as the shell. At the fusion temperature, the core goes through a phase shift. Within its fusion temperature, the core of NEPCM nanoparticles can undergo a phase shift to a liquid state and absorb or release energy in the form of latent heat.

### 2.1. Mathematical Modeling and Equations

As mentioned before, the buoyancy force (due to the temperature difference of the walls) and the moving upper wall produce the natural and forced convection currents, respectively. As a result of such a combination, one gets mixed convection. It is necessary to make some assumptions in order to predict the heat transfer behavior of NEPCMs when they are exposed to mixed convection. In this context, it is assumed that the distribution of NEPCMs throughout the combination of a base fluid and NEPCMs is consistent and stable. The thermophysical characteristics are unaffected by temperature in any way, with the exception of the density, which may be approximated using the Boussinesq approximation.

In addition, it is presumed that the nanoparticles and the base fluid are in a state of local thermal equilibrium that the flow is steady, two-dimensional, and incompressible.

In the sequel, we present the continuity, momentum, and energy equations in the x-y coordinates [35]:

(1) $$\frac{\partial u}{\partial x}+\frac{\partial v}{\partial y}=0$$

(2) $$\frac{\rho_{bf}}{\varepsilon }\frac{\partial u}{\partial t}+\frac{\rho_{bf}}{\varepsilon^{2}}\left(u\frac{\partial u}{\partial x}+v\frac{\partial u}{\partial y}\right)=-\frac{\partial p}{\partial x}+\frac{\mu_{bf}}{\varepsilon }\left(\frac{\partial^{2}u}{\partial x^{2}}+\frac{\partial^{2}u}{\partial y^{2}}\right)-\frac{\mu_{bf}}{K}u$$

(3) $$\begin{array}{ll} \frac{\rho_{bf}}{\varepsilon }\frac{\partial v}{\partial t}+\frac{\rho_{bf}}{\varepsilon^{2}}(u\frac{\partial v}{\partial x} & +v\frac{\partial v}{\partial y})\\ & =-\frac{\partial p}{\partial y}+\frac{\mu_{bf}}{\varepsilon }\left(\frac{\partial^{2}v}{\partial x^{2}}+\frac{\partial^{2}v}{\partial y^{2}}\right)+g\rho_{bf}\beta_{bf}\left(T-T_{c}\right)-\frac{\mu_{bf}}{K}v-\frac{\sigma {B_{0}}^{2}v}{\rho } \end{array}$$

(4) $$\left(u\frac{\partial }{\partial x}\left({\left(\rho C_{P}\right)}_{m}T\right)+v\frac{\partial }{\partial y}\left({\left(\rho C_{P}\right)}_{m}T\right)\right)=k_{m}\left(\frac{\partial^{2}T}{\partial x^{2}}+\frac{\partial^{2}T}{\partial y^{2}}\right).$$

The velocity components in the *x* and *y* directions are given by *u* and *v* in the above equations. In addition, the pressure, temperature, and acceleration due to gravity in the y-direction are given by *P*, *T*, and g, respectively. The last term in Equation (3) is the buoyant Boussinesq source term. The NEPCMs act through the advection term that appears in the energy conservation equation (Equation (4)). The impact of the NEPCM nanoparticles, as well as their effects on the overall performance of the problem, will be discussed later. Boundary conditions are required for the above coupled equations. As mentioned earlier, the walls of the cavity have a constant temperature of *T_c_*, whereas the temperature of the lower wall is given by a constant temperature of *T_h_*. We define the following dimensionless parameters, which leads in return to the dimensionless governing equations given below.

(5) $$\begin{array}{cc} \left(X,Y\right)=\left(\frac{x}{L},\frac{y}{L}\right),\left(U,V\right)=\left(\frac{u}{L},\frac{v}{L}\right) & \end{array}$$

(6) $$\theta =\frac{T-T_{c}}{T_{h}-T_{c}},P=\frac{p}{\rho_{bf}U^{2}}$$

(7) $$\frac{\partial U}{\partial X}+\frac{\partial V}{\partial Y}=0$$

(8) $$\left(\frac{\rho_{m}}{\rho_{bf}}\right)\left(U\frac{\partial U}{\partial X}+V\frac{\partial U}{\partial Y}\right)=-\frac{\partial P}{\partial X}+\frac{1}{{Re}_{bf}}\left(\frac{\mu_{m}}{\mu_{bf}}\right)\left(\frac{\partial^{2}U}{\partial X^{2}}+\frac{\partial^{2}U}{\partial Y^{2}}\right)-\frac{Pr}{Da}U$$

(9) $$\begin{array}{ll} \left(\frac{\rho_{m}}{\rho_{bf}}\right)(U\frac{\partial V}{\partial X}+ & V\frac{\partial V}{\partial Y})\\ & =-\frac{\partial P}{\partial Y}+\frac{1}{Re_{bf}}\left(\frac{\mu_{m}}{\mu_{bf}}\right)\left(\frac{\partial^{2}V}{\partial X^{2}}+\frac{\partial^{2}V}{\partial Y^{2}}\right)+\frac{Gr_{bf}}{{Re}_{bf}^{2}}\left(\frac{(\rho \beta {)}_{m}}{(\rho \beta {)}_{bf}}\right)\theta -\frac{\sigma_{m}}{\sigma_{bf}}Ha^{2}\;V-\frac{P_{r}}{Da}V \end{array}$$

(10) $$\left(U\frac{\partial }{\partial X}\left(Cr\theta \right)+V\frac{\partial }{\partial Y}\left(Cr\theta \right)\right)=\frac{1}{{Re}_{bf}Pr_{bf}}\left(\frac{k_{m}}{k_{bf}}\right)\left(\frac{\partial^{2}\theta }{\partial X^{2}}+\frac{\partial^{2}\theta }{\partial Y^{2}}\right)$$

The dimensionless numbers are expressed as follows [36]:

(11) $$v_{bf}=\frac{\mu_{bf}}{\rho_{bf}},\alpha_{bf}=\frac{k_{bf}}{{\left(\rho c_{p}\right)}_{bf}},Pr=\frac{v_{bf}}{\alpha_{bf}},Re_{bf}=\frac{UL}{v_{bf}},\;Ha=LB\sqrt{\frac{\sigma_{nf}}{\mu_{nf}}}\;\mathrm{and}\;Gr_{bf}=\frac{g\beta_{bf}\Delta Tr^{3}}{v_{bf}^{2}},$$

where $\;v_{bf},\;\alpha_{bf},\;Pr,\;{Re}_{bf}$ , and $Gr_{bf}$ represent the kinematic viscosity, thermal diffusion, Prandtl, Reynolds, and Grashof numbers, respectively [34]. Moreover, the dimensionless constant in the energy equation is the ratio between the heat capacity of the mixture and that of the base fluid (*Cr*) [32].

(12) $$Cr=\frac{{\left(\rho C_{P}\right)}_{m}}{{\left(\rho C_{P}\right)}_{bf}}=1-\varphi +\lambda \varphi +\frac{\varphi }{\chi }f$$

The subscripts  m and bf in Equation (12) stand for the nanoliquid mixture and base liquid, respectively. The specific heat capacity and density of liquid ${\left(\rho C_{P}\right)}_{bf}$ are assumed to be constants. Nevertheless, for certain materials of the NEPCMs and the base liquid, the values of the specific heat capacity of the mixture are a function of the amount of latent heat of the NEPCMs core, the volume fraction of the NEPCMs, and the temperature $\left(f\right).$ The variability relative to the volume fraction is shown by φ , whereas the variability associated with the latent heat of the NEPCM core is shown by χ . The quantity λ is calculated by [35]:

(13) $$\lambda =\frac{\left(C_{p_{c,l}}+lC_{p_{s}}\right)\rho_{c}\rho_{s}}{\left(\rho_{s}+l\rho_{c}\right){\left(\rho C_{P}\right)}_{bf}}$$

Clearly, λ is the ratio of the heat capacity of NEPCMs in the liquid phase to the base liquid. The subscripts c, l , and s stand for core, liquid, and sell, respectively. The core-shell weight ratio is given by l the coefficient and equals  0.447 . Moreover, the quantity χ in Equation (12) is calculated using the following [36].

(14) $$\chi =\frac{C_{P,bf}}{h_{sf}/T_{Mr}}\frac{\rho_{bf}\left(\rho_{s}+l\rho_{c}\right)}{\left(\rho_{s}\rho_{c}\right)}$$

where $h_{sf}$ is the latent heat of the core and $T_{Mr}$ is the melting temperature range of the core. Note that χ is the ratio of growth in the base liquid temperature to the energy stored as latent heat in the core [36]. In Equation (12), the quantity f is the dimensionless fusion function given by [35]:

(15) $$f=\frac{\pi }{2}\sin\left(\frac{\pi }{\delta }\left(\theta -\theta_{f}+\frac{\delta }{2}\right)\right)\times \left\{\begin{array}{l} 0\;\mathrm{if}\;\theta <\theta_{f}-\frac{\delta }{2}\\ 1\;\mathrm{if}\;\theta_{f}-\frac{\delta }{2}<\theta <\theta_{f}+\frac{\delta }{2}\\ 0\;\mathrm{if}\;\theta <\theta_{f}+\frac{\delta }{2} \end{array}\right.$$

where $\delta =\frac{T_{Mr}}{\Delta T}$ , and $\theta_{f}=\frac{T_{f}-T_{C}}{\Delta T}$ .

The parameters δ and $\theta_{f}$ stand for the dimensionless internal fusion and fusion temperature, respectively. Note that δ represents the thickness of the melting zone. Going back to Equation (15), one can see that if the nanoliquid temperature is larger than the NEPCM core melting temperature $\left(T>T_{f}+\frac{T_{Mr}}{2}\right)$ or smaller than the core solidification temperature $\left(T<T_{f}-\frac{T_{Mr}}{2}\right)$ , then the last term of Equation (12) becomes zero, and therefore the values of *Cr* are reduced. The melting temperature and the melting range also affect the values of *Cr*. Clearly, the heat transfer can be improved by changing the values of *Cr*. The substantial improvement in specific heat capacity when utilizing NEPCMs as opposed to conventional nanoparticles is the main advantage here. The coefficient of *f* appears in Equation (12) and is the amplitude of the change in specific heat capacity in the two-phase state. In comparison with Equation (15), it can be seen that the latent heat of the NEPCMs core *hsf* also has effects on the quantity *Cr*.

The rate of heat transfer can be computed using the Nusselt number as [35,36].

(16) $${Nu}_{Ave}=-\frac{1}{L}\int_{0}^{L}\frac{\partial \theta }{\partial n}dS$$

We mention here that the studied work was achieved in a permanent simulation (steady state) because the physical phenomena of the fluid in the studied range produces phenomena that are not related to the time Criterion.

### 2.2. The Thermophysical Properties

The NEPCM consists of an n-nonadecane core and a polyurethane shell. For thermal energy storage, the n-nonadecane is a favorable organic phase change material due to the fact that melting and solidification occur at a temperature of 31 °C. In addition, the latent heat of fusion of 156.07 kJ/kg and the latent heat of solidification of 164.99 kJ/kg. Hence, it can be used in buildings in order to store thermal energy. The operating temperature range and the amount of storable energy make such PCM feasible to be utilized in buildings [37]. On the other hand, the polyurethane shell has high strength, elevated elastic properties, smooth surface [36], and low Crystallinity [35]; therefore, it has been taken into consideration by many researchers. Moreover, such shell has no health or environmental issues related to the release of formaldehyde. Some previous experimental studies helped in extracting the thermophysical properties of water and the aforementioned NEPCM. The preparation of these nanoparticles for different applications was also studied and presented [36]. Considering the thermophysical properties of core and shell, the thermophysical properties of NEPCMs can be described as follows [35]:

(17) $$\rho_{n}=\frac{\left(1+l\right)\rho_{c}\rho_{s}}{\rho_{s}+l\rho_{c}}.$$

where *n* indicates the subscription of nanoparticles. The specific heat capacity of the nucleus can be presented in terms of a sinusoidal function whose argument is a function of temperature; the melting temperature *T_f_*, and the phase change temperature range *T_Mr_*, as described earlier. Its amplitude can also be seen as a function of the latent heat of the core *h_sf_*, and the specific heat capacity of the core in the liquid state *C_p_*. In addition, the specific heat capacity of the NEPCM core is *C_p_*, in the phase change temperature range [35]:

(18) $$\begin{array}{ll} C_{p,c} & =C_{pc,l}+\left\{\frac{\pi }{2}\left(\frac{h_{sf}}{T_{Mr}}-C_{pc,l}\right)\left(\sin\pi \frac{T-\left(T_{f}-T_{Mr}2\right)}{T_{Mr}}\right)\right\}\\ & \times \left\{\begin{array}{c} 0\;\mathrm{if}\;T<T_{f}-\frac{T_{Mr}}{2}\\ 1\;\mathrm{if}\;T_{f}-\frac{T_{Mr}}{2}<T<T_{f}+\frac{T_{Mr}}{2}\\ 0 \end{array}\right.\\ & \;\mathrm{if}\;T<T_{f}+\frac{T_{Mr}}{2} \end{array}$$

Below, we introduce the specific heat capacity and thermal expansion coefficient of NEPCM [37]:

(19) $$C_{p,n}=\frac{\left(C_{p,c}+lC_{s}\right)\rho_{c}\rho_{s}}{\left(\rho_{s}+l\rho_{c}\right)\rho_{n}}$$

(20) $$\beta_{n}=\beta_{c}+\left(\frac{\beta_{s}-\beta_{c}}{2}\right)\left(1-\frac{l\rho_{s}}{\rho_{c}}\right)$$

The thermophysical properties of liquid, NEPCM nanoparticles, NEPCM volume fraction, and the water-NEPCM mixture are given in Table 1. The thermophysical equations of the mixture, including density, specific heat capacity, and thermal expansion coefficient, are given by the following equations [35,38].

(21) $$\rho_{m}=\left(1-\varphi \right)\rho_{bf}+\varphi \;\;\;\;\;\;\;\;\;\;\;\;\;\;\;\;\;\;\;\;\;\;\;\;\;\;\;\;\;\;$$

(22) $$\rho_{n}\;\mathrm{and}\;C_{P,m}=\frac{\left(1-\varphi \right)\rho_{f}C_{P,bf}+\varphi \rho_{n}C_{P,n}}{\rho_{m}}$$

(23) $$\;\beta_{m}=\frac{\left(1-\varphi \right)\rho_{bf}\beta_{bf}+\varphi \rho_{n}\beta_{n}}{\rho_{m}}$$

In all equations, the subscripts m, n, c, and s stand for the mixture, NEPCM, core, and shell, respectively. The performed simulation was conducted with a constant volume fraction of 0.035. Hence, as indicated by [35], the thermal conductivity and dynamic viscosity of the mixture at 303 K are equal to 122 × 10^−5^ kg/m.s and 0.7 W/m.K, respectively. The thermophysical properties of the materials that are used are given in Table 1.

**Figure 2:**
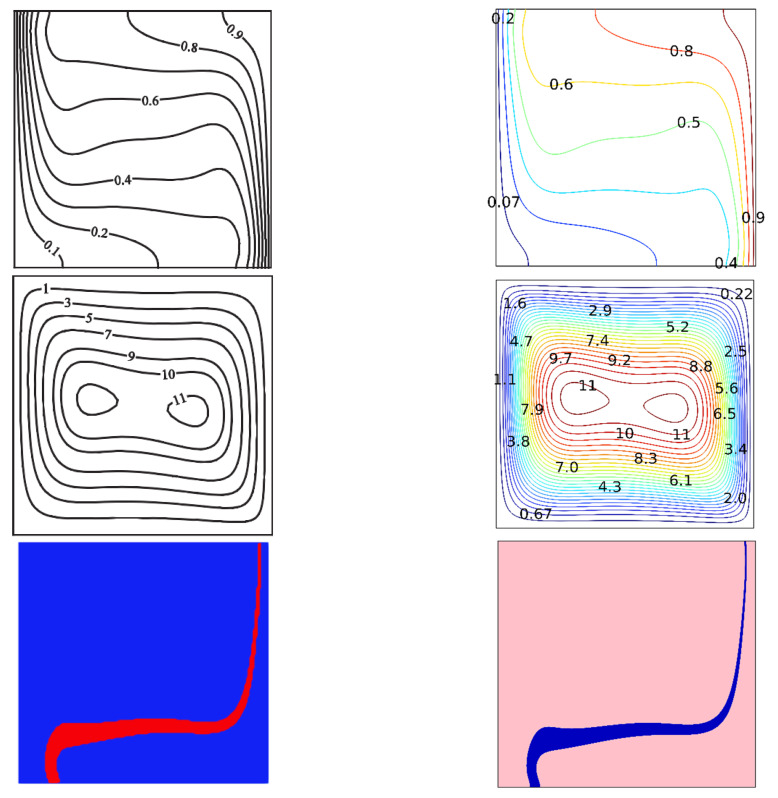
Comparison of current work with that of Mohammad Ghalambaz et al. reprinted/adapted with permission from Ref. [35]. 2022, Elsevier.

**Validation:** The results of the previous study were confirmed using numerical analysis. Despite the fact that there are different numerical techniques that can be employed to solve the differential equations appear in different applications (see, e.g. [41,42,43,44]), the Galerkin Finite Element has been applied here to simulate the model’s numerical solutions. Figure 2 shows a comparison between the outcomes from the model employed in the current investigation and those shown in Ghalambaz et al. [35]. 

Table 2 shows the average Nusselt number results for various grid sizes for the greatest values of interest parameters and various power-law fluid indices (Ha = 0, Re= 500). In the following computations, several elements of 23,522 were used.

## 3. Results and Discussion

We present in this section the results that were obtained in order to clarify the thermal and dynamic composition of a homogeneous suspension of NEPCM particles and water. This new type of suspension is studied in a chamber with a lid and trapezoidal geometry with a zigzag bottom. The questions to be studied are:

- The value of Reynolds number (Re) is in the range of 1–500. This number (Re) expresses the velocity of the movement of the top of the container.
- The value of Hartmann’s number (Ha) is in the range of 0–100. This element (Ha) determines the magnetic field acting on the space from the outside.
- Finally, the value of the Darcy number (Da) was studied in the range of 10^−5^–10^−2^. The permeability of the medium is determined by this number (Da).
- The shape of the soil changed by changing the number of ripples in the range of 1–4.

We find that the suspension (water + NEPCM particles) moves due to two factors, namely the horizontal movement of the top of the chamber and the force of thermal buoyancy (Gr = 1000). Understanding and deducing the dynamic behavior of the suspension flow and its direct influence on the heat transfer process is primarily done by analyzing the contours of the isotherms, heat capacity and trajectories. The purpose of adding NEPCM particles is to enhance the thermal properties of the water (heat capacity and conductivity).

**Figure 3:**
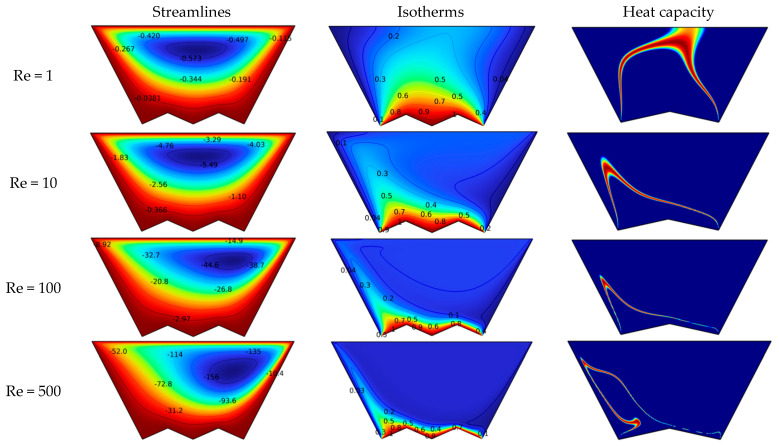
Effect of Reynolds number of the isotherm, heat capacity, and streamlines for Ha = 0, Da = 10^−2^, and N = 2.

To understand the effect of the velocity of motion of the wall, Figure 3 shows the incoming changes in the trajectories, dimensionless temperature, and heat capacity as a function of Reynolds number for Ha = 0, Da = 10^−2^, and N = 2. Figure 3 shows that the motion of the wall Creates a circular flow in space with its center near the moving wall. On the other hand, if we increase the value of Re, the velocity spread of the space increases, which also causes the vortex to move to the right side of the space. It can be concluded that the velocity of the rotating flow in the space increases as Re increases. For the dimensionless temperature, we find that there is an accumulation of isotherms near the bottom as Re increases. This accumulation is an indication of the high value of the temperature gradient. That is, the thermal activity interacts positively with respect to Re. Moreover, it is observed that the concentration of isotherms on the right-hand side of the soil is better than on the left-hand side, which means that the heat transfer is better on the right side of the soil. The heat capacity contours reflect the same behavior derived from the isotherms and streamlines. First, we note that the red line indicates the regions where the change in the physical state of the NEPCM particles occurs. We note that the red line follows the motion of the flow. The high-temperature gradient makes the line narrow and close to the bottom on the right side.

**Figure 4:**
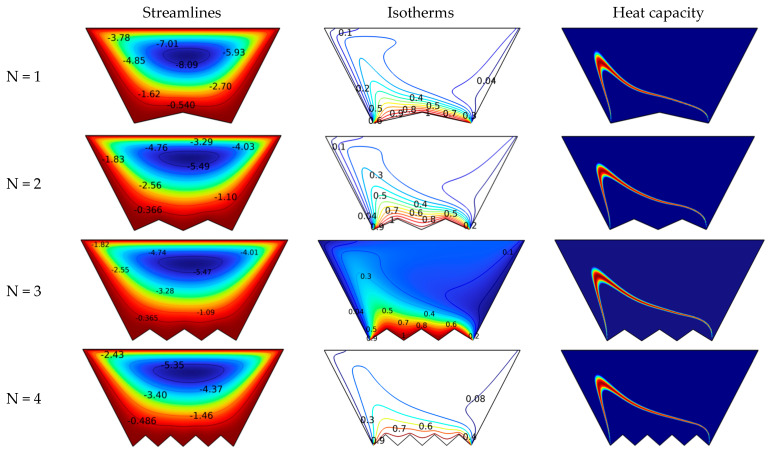
Effect of the number of zigzags on the isotherm, heat capacity, and streamlines for Ha = 0, Da = 10^−2^, and Re = 10.

Figure 4 shows the effects of soil shape on suspension stirring and thermal pattern. Therefore, Figure 4 demonstrates the effects of increasing the number of zigzag lines on the streamlines, isotherms, and heat capacity for Ha = 0, Da = 10^−2^, and Re = 10. When examining the streamlines, it is clear that the greater the number of zigzag lines on the bottom, the slower the velocity of the suspension flow. This is normal because the more zigzag lines the bottom has, the more it hinders the flow motion, which leads to a decrease in its velocity. As the velocity gradually decreases, the dimensionless temperatures also show a decrease in isothermal density near the ground, i.e., a decay in heat transfer in the form of the zigzag number. The same observations can be derived from the heat capacity diagrams. That is, the larger the zigzag number, the greater the distance of the red bar from the ground is.

**Figure 5:**
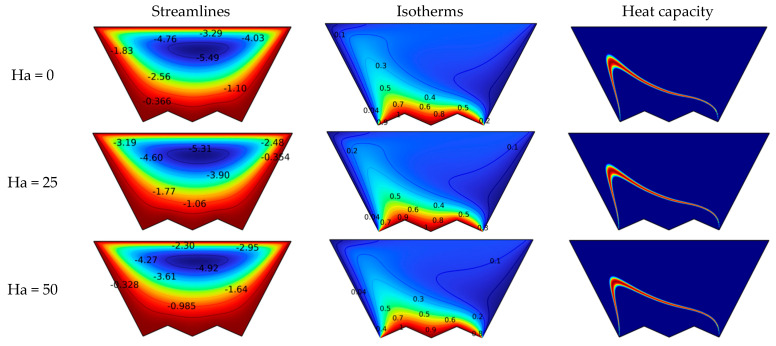

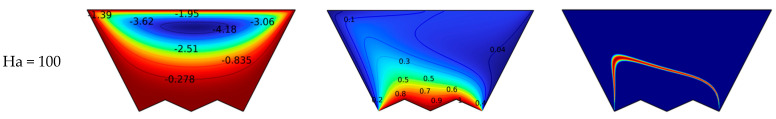
Effect of the number of Ha on the isotherm, heat capacity, and streamlines for Da = 10^−2^ and N = 2.

Figure 5 shows the effect of magnetic field strength (Ha = 0–100) on the motion of the suspension and its thermal pattern for N = 2, Da = 10^−2^, and Re = 10. As the suspension moves in a magnetic field, a force, the Lorentz force, is generated that impedes the motion of the suspension in the chamber. Therefore, the streamlines show a decrease in the velocity of the suspension flow as the number Ha grows. In addition, the size of the main vortex formed decreases as the value of Ha increases. The isotherms and heat capacity contours also confirm that the velocity decreases because the temperature gradient near the bottom decreases, and the distance between the bottom and the heat capacity line increases with increasing Ha.

**Figure 6:**
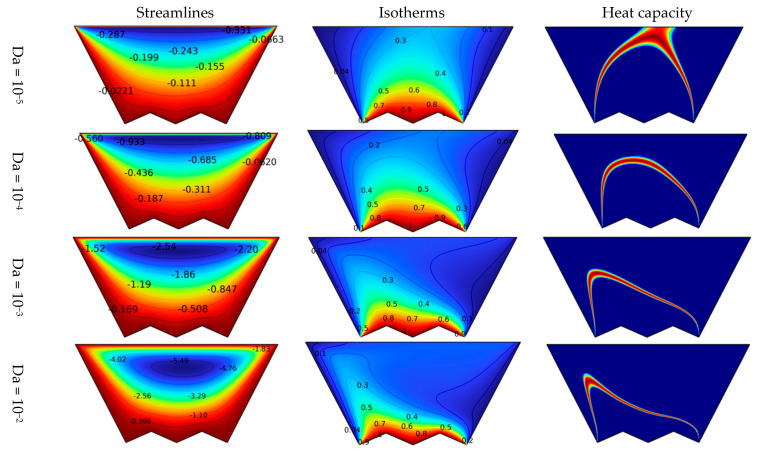
Effect of the number of Da on the isotherm, heat capacity, and streamlines for Ha = 0 and N = 2.

Figure 6 explains the evolution of the individual streamlines, heat capacity, and dimensionless temperature as a function of the Darcy number for N = 2, Re = 10, and Ha = 0. The value of the Darcy number expresses the permeability of the space, i.e., the larger this value, the better the permeability is. The streamlines show that the higher the Da number, the higher the velocity of the suspended particles and the more pronounced the development of the vortex is. The reason, of course, is the permeability of the space. That is, the greater the permeability, the more positive effect on the levitation is. In the contours of heat capacity and isotherms, there is a rise in the temperature gradient near the bottom, and the red line approaches the bottom because of the growth in the flow velocity inside the medium.

**Figure 7:**
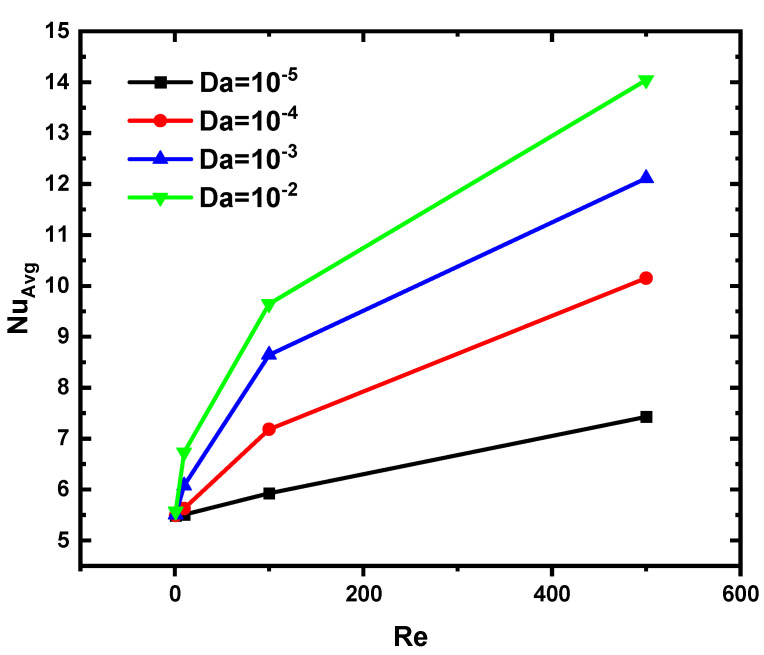
Variation of Nu versus Re and Da for N = 2 and Ha = 0.

Figure 7 shows the evolution of the Nu number of the hot soil surface as a function of Re and Da for N = 2 and Ha = 0. It can be noted that the higher the value of Re or Da, the higher the value of the Nu number is. The reason for this increase is that the higher the value of Da or Re, the better the flow velocity is, which increases the heat transfer, i.e., the value of the Nusselt number increases.

**Figure 8:**
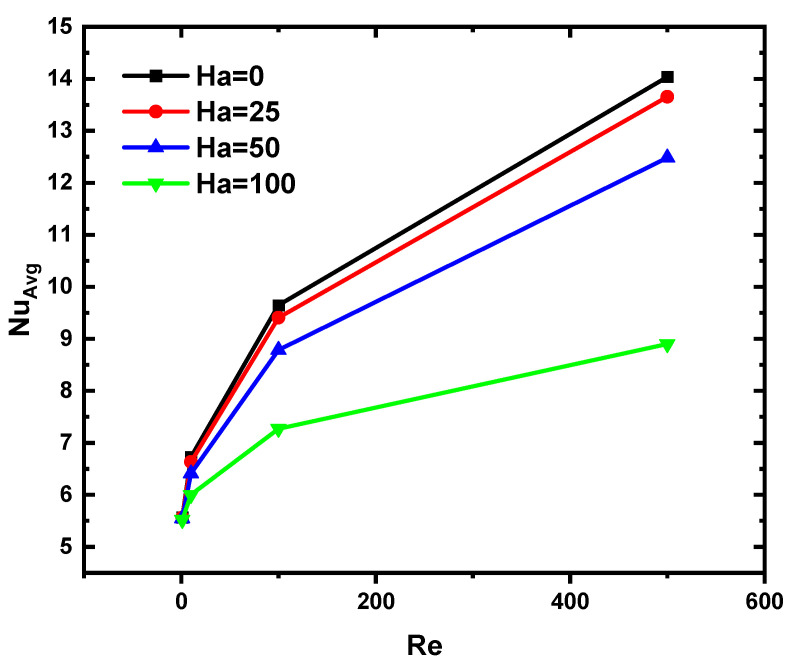
Variation of Nu versus Re and Ha for N = 2 and Da = 10^−2^.

The effects of the Ha and Re numbers on Nu for N = 2 and Da = 10^−2^ are shown in Figure 8. It can be observed that the higher the value of the Ha number, the lower the value of Nu is, which indeed, results from the Lorentz force, that prevents the movement of the suspension and, as a result, causes a reduction in the thermal activity between the fluid and the hot wall.

**Figure 9:**
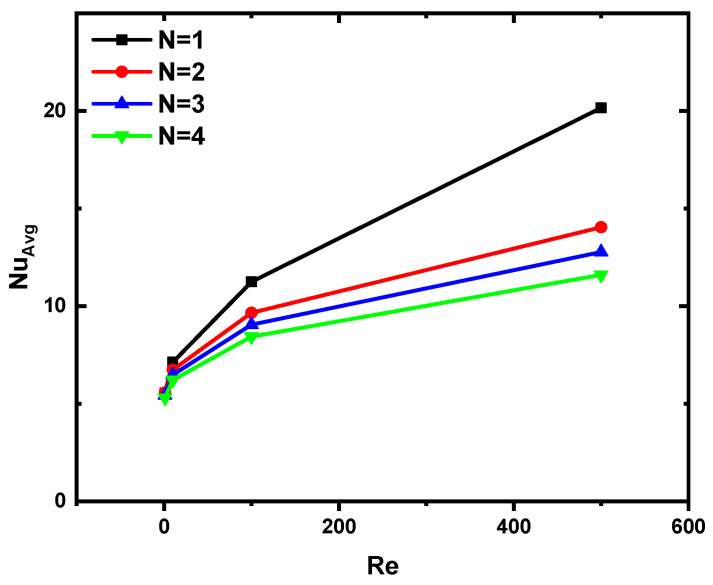
Variation of Nu versus Re and N for Ha = 0 and Da = 10^−2^.

In Figure 9, we show the bottom wall shape’s effect on the suspension flow’s thermal performance. As the number N illustrates the effect of the number of zigzag lines of the soil on the number of Nu for Da = 10^−2^ and without the MHD effect (Ha = 0). It can be observed that the higher the number of bottom ripples, the lower the value of the Nusselt number is, i.e., the thermal activity decreases, which is due to the shape of the bottom, i.e., the increase in ripples makes it more difficult for the flow to stick to the bottom, reducing the heat transfer and thus decreasing the value of the Nusselt number.

Through our analysis of all figures, we can adopt a general idea, which is that the better and easier the fluid movement, the greater the thermal transfer.

## 4. Conclusions

In this work, we simulate numerically the mixed convection of NEPCM confined in a trapezoidal lidded cavity saturated with porous media and subject to a magnetic field. While the upper cavity wall is insulated and moves at a constant speed, we let the lower one be zigzagged and maintained at a cold temperature. In addition, the two sloping side walls are maintained at a hot temperature. The effects of Reynolds number (Re = 1, 10, 100, 500), zigzag number of the lower wall (N = 1, 2, 3, 4), Hartmann number (Ha = 0, 25, 50, 100), and Darcy number (Da = 10^−5^, 10^−4^, 10^−3^, 10^−2^) are presented and analyzed. From the study results, we demonstrate the following conclusions:

- Higher values of Re increase the velocity of the flow, resulting in better temperature distribution.
- An increase in the zigzag number of the lower wall hinders the flow velocity; this leads to a limitation of the temperature distribution and a decrease in the heat capacity.
- Increasing the value of Ha (increasing the intensity of the magnetic field) had a negative effect on the heat transfer inside the cavity because the magnetic field inhibits the flow velocity inside the cavity.
- Heat transfer inside the cavity lid improved at higher values of Darcy number because the permeability of the cavity increased with increasing Da.
- At the highest Re, an increase in Da resulted in a 100% increase in Nu, while an increase in N and Ha resulted in a 45% and 38% decrease in Nu, respectively.

## Figures and Tables

**Table 1:** The thermophysical properties of the materials that are used at 303 K [32,33,34,35,36,37,38,39,40].

Material Property	$\rho \left(\frac{kg}{m^{3}}\right)$	$C_{p}\left(\frac{J}{kgK}\right)$	$\beta \times 10^{-5}\left(\frac{1}{K}\right)$	$k\left(\frac{W}{mK}\right)$	$\mu \times 10^{-6}\left(\frac{\mathrm{kg}}{\mathrm{ms}}\right)$
Water (Base fluid).	955.6	4180	21	0.615	797
Nonadecane (core)	786	1317	50	0.19	-
Polyurethane (shell)	721	2037	17.3	0.025	-

**Table 2:** Grid sensitivity check (Ha = 0, φ = 0.04, n = 1, Re = 100).

	959	2049	3376	5165	23,522	88,044
$\psi_{\max}$	0.0407	0.039	0.036	0.035	0.035	0.035
Nu_avg_	14.12	0.039	14.07	14.04	14.02	14.02

## Data Availability

Not applicable.

## Nomenclatures

**Latin Letters**		**Greek symbols**	
$C_{r}$	the ratio of heat capacity of mixture to water	α	$\mathrm{thermal}\;\mathrm{diffusivity}\;\left(m^{2}/s\right)$
$C_{p}$	specific heat capacity(J/kgK)	δ	thickness of melting zone
f	fusion function	θ	non-dimensional temperature
g	$\mathrm{gravitational}\;\mathrm{acceleration}\;\left(m/s^{2}\right)$	λ	the ratio of heat capacity of NEPCM to water
Gr	Grashof number	μ	dynamics viscosity (kg/ms)
$h_{sf}$	latent heat of core (J/kg)	v	$\mathrm{kinematic}\;\mathrm{viscosity}\;\left(m^{2}/s\right)$
k	thermal conductivity (W/mK)	$\vartheta_{s}$	volume ratio of core to shell
L	length of the cavity (m)	ρ	$\mathrm{density}\;\left(kg/m^{3}\right)$
l	weight ratio of core to shell	χ	the intensity of stored energy in the core
$Nu_{\mathrm{Avg}}$	average Nusselt number		
p	pressure (Pa)		
Pr	Prandtl number	Subscript	
Re	Reynolds number	f	fusion
T	temperature (K)	n	nanoparticle
$T_{Mr}$	melting temperature range (K)	m	mixture of water-NEPCM
$T_{f}$	fusion temperature (K)	l	liquid phase
V	velocity vector (m/s)	s	sell of the NEPCM
