# Correction: Abderrahmane et al. 2D MHD Mixed Convection in a Zigzag Trapezoidal Thermal Energy Storage System Using NEPCM. *Nanomaterials* 2022, *12*, 3270

**DOI:** 10.3390/nano16020081

**Published:** 2026-01-07

**Authors:** Aissa Abderrahmane, Obai Younis, Mohammad Al-Khaleel, Houssem Laidoudi, Nevzat Akkurt, Kamel Guedri, Riadh Marzouki

**Affiliations:** 1 Laboratoire de Physique Quantique de la Matière et Modélisation Mathématique (LPQ3M), University of Mascara, Mascara 29000, Algeria; a.aissa@univ-mascara.dz; 2 Department of Mechanical Engineering, College of Engineering in Wadi Addwasir, Prince Sattam Bin Abdulaziz University, Al-Kharj 11942, Saudi Arabia; 3 Department of Mathematics, Khalifa University, Abu Dhabi 127788, United Arab Emirates; 4 Department of Mathematics, Yarmouk University, Irbid 21163, Jordan; 5 Laboratory of Sciences and Marine Engineering (LSIM), Oran 31000, Algeria; 6 Department of Mechanical Engineering, Munzur University, 62000 Tunceli, Turkey; 7 Mechanical Engineering Department, College of Engineering and Islamic Architecture, Umm Al-Qura University, P.O. Box 5555, Makkah 21955, Saudi Arabia; 8 Chemistry Department, College of Science, King Khalid University, Abha 61413, Saudi Arabia; 9 Chemistry Department, Faculty of Sciences of Sfax, University of Sfax, Sfax 3038, Tunisia

There were errors in the original publication [1].

In Abstract and Introduction, any reference to “heat transfer (HT) irreversibility,” “fluid friction (FF) irreversibility,” or “Bejan number” is erroneous and should be removed. Any reference to “wave number” should be corrected to “zigzag number,” which is the relevant parameter in the study. The corrected Abstract and the corrected sentence in the last paragraph of the Introduction appear below, respectively.

In a magnetic field, two-dimensional (2D) mixed convection is investigated within a zigzagged trapezoidal chamber. The lower side of the trapezoidal chamber is irregular, in particular, a zigzagged wall with different zigzag numbers N. The fluid particles move in the room due to the motion of the upper wall, while the porosity-enthalpy approach represents the melting process. The thermal parameters of the fluid are enhanced by what is called a nano-encapsulated phase change material (NEPCM) consisting of polyurethane as the shell and a nonadecane as the core, while water is used as the base fluid. In order to treat the governing equations, the well-known Galerkin finite element method (GFEM) is applied. In addition, the heat transfer (HT) and the dynamic behavior of flow are treated in terms of average Nusselt number and contours of streamlines. The main results show that the melt band curve of heat capacity behaves almost parabolically at smaller values of Reynolds number (Re) and larger values of Hartmann number (Ha). Moreover, minimizing the zigzag number is better in order to obtain a higher heat transfer rate.

In addition, a comparative study of the heat transfer and the dynamic behavior of flow in terms of average Nusselt number and contours of streamlines has been presented.

In Section 2.1. Mathematical Modeling and Equations, Paragraph 3 and Paragraph 4, the term of porosity must be added to the momentum equations. The Equations (2), (3), (8) and (9) should be updated to the following:

(2) $$\frac{\rho_{bf}}{\varepsilon }\frac{\partial u}{\partial t}+\frac{\rho_{bf}}{\varepsilon^{2}}\left(u\frac{\partial u}{\partial x}+v\frac{\partial u}{\partial y}\right)=-\frac{\partial p}{\partial x}+\frac{\mu_{bf}}{\varepsilon }\left(\frac{\partial^{2}u}{\partial x^{2}}+\frac{\partial^{2}u}{\partial y^{2}}\right)-\frac{\mu_{bf}}{K}u$$

(3) $$\begin{array}{ll} \frac{\rho_{bf}}{\varepsilon }\frac{\partial v}{\partial t}+\frac{\rho_{bf}}{\varepsilon^{2}}(u\frac{\partial v}{\partial x} & +v\frac{\partial v}{\partial y})\\ & =-\frac{\partial p}{\partial y}+\frac{\mu_{bf}}{\varepsilon }\left(\frac{\partial^{2}v}{\partial x^{2}}+\frac{\partial^{2}v}{\partial y^{2}}\right)+g\rho_{bf}\beta_{bf}\left(T-T_{c}\right)-\frac{\mu_{bf}}{K}v-\frac{\sigma {B_{0}}^{2}v}{\rho } \end{array}$$

(8) $$\left(\frac{\rho_{m}}{\rho_{bf}}\right)\left(U\frac{\partial U}{\partial X}+V\frac{\partial U}{\partial Y}\right)=-\frac{\partial p}{\partial X}+\frac{1}{{Re}_{bf}}\left(\frac{\mu_{m}}{\mu_{bf}}\right)\left(\frac{\partial^{2}U}{\partial X^{2}}+\frac{\partial^{2}U}{\partial Y^{2}}\right)-\frac{Pr}{Da}U$$

(9) $$\begin{array}{ll} \left(\frac{\rho_{m}}{\rho_{bf}}\right)(U\frac{\partial V}{\partial X}+ & V\frac{\partial V}{\partial Y})\\ & =-\frac{\partial p}{\partial Y}+\frac{1}{Re_{bf}}\left(\frac{\mu_{m}}{\mu_{bf}}\right)\left(\frac{\partial^{2}V}{\partial X^{2}}+\frac{\partial^{2}V}{\partial Y^{2}}\right)+\frac{Gr_{bf}}{{Re}_{bf}^{2}}\left(\frac{(\rho \beta {)}_{m}}{(\rho \beta {)}_{bf}}\right)\theta -\frac{\sigma_{m}}{\sigma_{bf}}Ha^{2}\;V-\frac{P_{r}}{Da}V \end{array}$$

In Section 2.1. Mathematical Modeling and Equations, Paragraph 4, Paragraph 5 and Paragraph 9, there is no cylinder inside the cavity, and any reference to a “cylinder” or “diameter D” is incorrect and should be disregarded. The pressure in Equation (6), the Reynolds Number in Equation (11) and the definition for the average Nusselt number at the zigzag wall in Equation (16) should be updated to the following:

(6) $$\theta =\frac{T-T_{c}}{T_{h}-T_{c}},P=\frac{p}{\rho_{bf}U^{2}}$$

(11) $$\begin{array}{c} v_{bf}=\frac{\mu_{bf}}{\rho_{bf}},\alpha_{bf}=\frac{k_{bf}}{{\left(\rho c_{p}\right)}_{bf}},Pr=\frac{v_{bf}}{\alpha_{bf}},Re_{bf}=\frac{UL}{v_{bf}},\\ Ha=LB\sqrt{\frac{\sigma_{nf}}{\mu_{nf}}}\;\mathrm{and}\;Gr_{bf}=\frac{g\beta_{bf}\Delta Tr^{3}}{v_{bf}^{2}}, \end{array}$$

(16) $${Nu}_{Ave}=-\frac{1}{L}\int_{0}^{L}\frac{\partial \theta }{\partial n}dS$$

The authors state that the scientific conclusions are unaffected. This correction was approved by the Academic Editor. The original publication has also been updated.
